# Heterogeneous-IRS-Assisted Millimeter-Wave Systems: Element Position and Phase Shift Optimization

**DOI:** 10.3390/s25154688

**Published:** 2025-07-29

**Authors:** Weibiao Zhao, Qiucen Wu, Hao Wei, Dongliang Su, Yu Zhu

**Affiliations:** 1 Key Laboratory for Information Science of Electromagnetic Waves (MoE), School of Information Science and Technology, Fudan University, Shanghai 200433, China; zhaowb22@m.fudan.edu.cn (W.Z.); qcwu21@m.fudan.edu.cn (Q.W.); 2 ZTE Corporation, Shanghai 201203, China; wei.hao@zte.com.cn (H.W.); su.dongliang@zte.com.cn (D.S.)

**Keywords:** heterogeneous intelligent reflecting surface, millimeter-wave system, particle swarm optimization, manifold optimization, Cauchy–Schwarz bound

## Abstract

Intelligent reflecting surfaces (IRSs) have attracted extensive attention in the design of future communication networks. However, their large number of reflecting elements still results in non-negligible power consumption and hardware costs. To address this issue, we previously proposed a green heterogeneous IRS (HE-IRS) consisting of both dynamically tunable elements (DTEs) and statically tunable elements (STEs). Compared to conventional IRSs with only DTEs, the unique DTE–STE integrated structure introduces new challenges in optimizing the positions and phase shifts of the two types of elements. In this paper, we investigate the element position and phase shift optimization problems in HE-IRS-assisted millimeter-wave systems. We first propose a particle swarm optimization algorithm to determine the specific positions of the DTEs and STEs. Then, by decomposing the phase shift optimization of the two types of elements into two subproblems, we utilize the manifold optimization method to optimize the phase shifts of the STEs, followed by deriving a closed-form solution for those of the DTEs. Furthermore, we propose a low-complexity phase shift optimization algorithm for both DTEs and STEs based on the Cauchy–Schwarz bound. The simulation results show that with the tailored element position and phase shift optimization algorithms, the HE-IRS can achieve a competitive performance compared to that of the conventional IRS, but with much lower power consumption.

## 1. Introduction

With the rapid development of wireless communication networks, the number of mobile devices has grown exponentially, leading to increasing demand for high-data-rate services. Millimeter-wave (mmWave) communication, as a key enabling technology, provides an abundant available bandwidth in high-frequency ranges, making it a promising solution to meet these demands. However, the severe path loss and high susceptibility to blockages inherent in mmWave signals still remain critical challenges that hinder their practical implementation [1,2,3,4].

To tackle these issues, intelligent reflecting surfaces (IRSs) have recently been introduced into mmWave communication systems [5,6]. An IRS is generally composed of a large number of reconfigurable elements, each capable of reflecting the incident signal with a tunable phase shift. By smartly tuning the phase shifts of all reconfigurable elements adaptively according to the channel state information (CSI), the reflecting signals from the IRS can enhance the desired signal power, extend the coverage, and suppress co-channel interference. This can significantly improve the spectrum efficiency, energy efficiency, and reliability of mmWave communication systems, without the need for deploying additional active base stations (BSs) or relays. For instance, IRSs can optimize wireless channels in sensor networks, enabling reliable communication between distributed sensors, even in challenging environments like urban areas with significant signal blockages. Furthermore, from an implementation perspective, IRSs have the features of being low profile and lightweight, allowing for easy installation on the walls or ceilings of buildings and their integration into existing cellular systems without requiring hardware changes to BSs/access points (APs) or user terminals. These appealing advantages have stimulated many research efforts in developing IRS-assisted wireless communication systems using different metrics, including maximizing spectral efficiency [7], energy efficiency [8], sum-rate of all users [9,10,11], and received power [12], as well as minimizing transmit power [13,14,15], etc.

However, many existing studies often focus on the performance improvement of IRSs, while the explicit power consumption model of IRSs has received little attention. In [16], the authors proposed the quantitative relationship between the dynamic power consumption of an IRS and the polarization mode, controllable bit resolution, and working status of the IRS, and then validated it via practical experimental results. The experimental results in [16] showed that 3600 positive–intrinsic–negative (PIN) diode-based 1-bit elements can consume up to 45 W of power. In [17], the authors further indicated that the static power consumption of IRSs, particularly the power loss caused by the control circuitry, increases with the expansion of the IRS size. This effect becomes particularly significant when the number of reconfigurable elements becomes large. Furthermore, in addition to the dynamic and static power consumption, the deployment of large IRS elements and the associated control circuitry poses significant challenges in complexity and high costs.

Motivated by the above challenges, we proposed a novel heterogenous IRS (HE-IRS) in [18] in order to better provide a trade-off between power consumption and system performance. In particular, a proportion of dynamically tunable elements (DTEs) in the conventional IRS are replaced by statically tunable elements (STEs). DTEs are subject to the constraint of quantized phase shifts due to the implementation of PIN diodes, while STEs can achieve near-infinite precision phase shifting control through meta-surface design [19,20,21,22,23]. Additionally, STEs do not require any circuitry for control and can be realized using completely passive physical structures, which means that they are predetermined and set during the manufacturing process, thereby avoiding power consumption. In [24], the authors proposed a tunable meta-surface that leverages PIN diodes to control the transmission and reflection phases, enabling multiple functionalities such as beamforming, anomalous reflection, and diffusion. Meanwhile, the authors in [25] proposed an electronically steerable array that achieves dual-functional beam scanning (frequency scanning and fixed-frequency electronic scanning) using varactor diodes in a leaky-wave antenna structure. Additionally, a hybrid IRS architecture was proposed in [26], which integrates both active and passive reflecting elements to enhance communication performance. In contrast, our proposed HE-IRS incorporates the following two distinct types of passive reflecting elements: DTEs and STEs. This unique design eliminates the need for active components, significantly reducing power consumption and hardware complexity. By combining DTEs, which offer dynamic phase adjustment, with STEs, which provide pre-designed fixed phase shifts, the HE-IRS achieves a more energy-efficient and cost-effective solution. These distinctive advantages make the HE-IRS particularly suitable for energy-sensitive and cost-constrained applications, such as green communication systems. However, it also introduces the issue of position optimization for both DTEs and STEs, while also requiring the design of phase shifts for DTEs with finite-precision phase shifts and STEs with infinite-precision phase shifts. This makes the optimization problem become a multi-variable, non-smooth, and non-convex problem.

To tackle these difficulties, we establish a problem for optimizing the element position and phase shifts of both DTEs and STEs in mmWave communication systems, aiming to maximize the average received power over a user angular space covered by the HE-IRS. To solve this problem, we first decouple the element position and phase shift optimization and solve the position optimization subproblem by a particle swarm optimization (PSO) algorithm. Then, we decompose the phase shifts of both the DTEs and STEs and alternately solve them. We utilize manifold optimization (MO) to optimize the phase shifts of the STEs and derive a closed-form solution for those of the DTEs. Furthermore, we propose a low-complexity phase design method based on the Cauchy–Schwarz bound (CSB) which does not require iteration. The simulation results illustrate that with the tailored element position and phase shift optimization algorithms, the HE-IRS can achieve a competitive performance compared to that of the conventional IRS, but with much lower power consumption, regardless of the large angular space, high controlling quantization, and large-scale arrays of IRSs.

The rest of this paper is organized as follows. In Section 2, we introduce the structure of the HE-IRS, illustrate the considered HE-IRS-assisted mmWave communication system, and formulate an optimization problem for optimizing the element position and phase shifts of the HE-IRS. Section 3 proposes an efficient algorithm for solving the non-smooth non-convex problem, where detailed algorithms for both the element position and phase shifts of the HE-IRS are discussed, and a low-complexity phase shift optimization is also presented in this section. We provide extensive simulation results in Section 4 and draw our conclusions in Section 5.

*Notation:* Boldface lower- and uppercase symbols denote vectors and matrices, respectively. $C^{m\times n}$ and $R^{m\times n}$ denote the set of m×n complex- and real-valued matrices, respectively. ${\left(\cdot \right)}^{*}$, ${\left(\cdot \right)}^{T}$, ${\left(\cdot \right)}^{H}$, and ${\left(\cdot \right)}^{-1}$ denote the conjugate, transpose, conjugate–transpose operations of a matrix or vector and the inversion of a matrix, respectively. $\mathrm{tr}\left(\cdot \right)$, ${\left\|\cdot \right\|}_{0}$, and $\left\|\cdot \right\|$ denote the trace, $l_{0}$-norm, and $l_{2}$-norm of a matrix, respectively. $E\left[\cdot \right]$ denotes the statistical expectation and R{⋅} represents the real part of a complex number. The imaginary element of a complex number is denoted by $j=\sqrt{-1}.$ $I_{N}$ denotes the N×N identity matrix and $1_{N\times 1}$ denotes the N×1 vector consisting of all ones. $diag\left(x\right)$ is a diagonal matrix with the entries of x on its main diagonal. $CN(0,\sigma^{2})$ denotes the circularly symmetric complex Gaussian distribution with a zero mean and covariance $\sigma^{2}$. ⊙ is the Hadamard product of two vectors or matrices.

## 2. HE-IRS Structure and System Model

### 2.1. HE-IRS Structure

Although the introduction of IRSs has many advantages for wireless communications, the huge number of reconfigurable elements can lead to prohibitive power consumption and system complexity. To deal with this problem, in [18], we proposed a novel HE-IRS, which can significantly reduce system complexity and power consumption.

**Figure 1:**
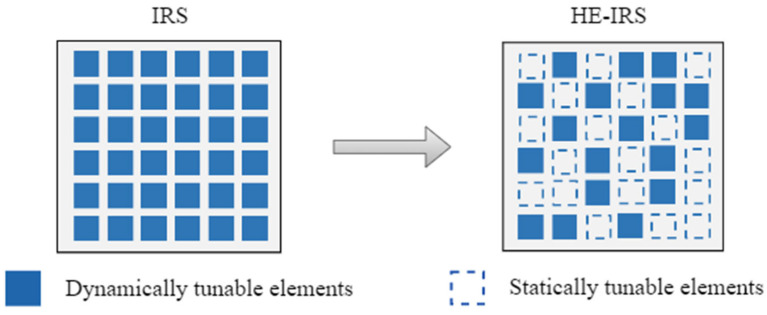
Illustration of the conventional IRS and the proposed HE-IRS structures.

Figure 1 illustrates the schematics of both the conventional IRS and the HE-IRS, which incorporate the following two distinct types of reflecting elements: dynamically tunable elements (DTEs) and statically tunable elements (STEs). DTEs provide discrete phase shifts for incident signals by employing PIN diodes or varactor diodes and are equipped with control circuits that enable dynamic adaptation to varying wireless environments. However, their phase shift resolution is typically limited, as practical IRS prototypes often adopt either 1-bit on–off phase shifters or 2-bit quadruple-level phase shifters [27]. In contrast, STEs consist of fixed physical structures without control circuits, making them static after deployment. This design significantly reduces hardware complexity and power consumption compared to DTEs. Constructed from meta-surfaces, such as Minkowski structures [21,22,23], STEs have been experimentally validated for millimeter-wave and terahertz applications, achieving excellent an phase-shifting capability through precise design, such as adjusting geometric parameters like loop width [23]. While DTEs offer dynamic reconfigurability based on instantaneous CSI, their phase shift resolution remains constrained. Conversely, STEs provide high-resolution phase shifts but are static. By combining DTEs and STEs, the HE-IRS architecture strikes a balance between passive beamforming performance, power consumption, and system complexity.

### 2.2. Scenario Description

**Figure 2:**
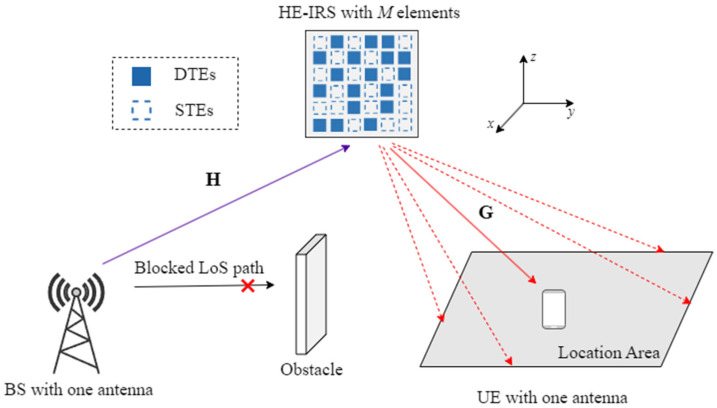
Diagram of an HE-IRS-aided single-user SISO wireless system.

In this paper, we consider a point–point single-input, single-output (SISO) mmWave communication system, as shown in Figure 2, where the direct link between the BS and the user is assumed to be blocked, and an HE-IRS composed of *M* reflecting elements is deployed to assist communication between the BS and the user. We assume that the line-of-sight (LoS) paths dominate in all channels, i.e., the BS–IRS channel and the IRS–User channel. Since the BS and HE-IRS are hardly moved once deployed, we assume that the LoS path of the BS–IRS channel is fixed. For the IRS–User channel, the user is randomly located in an area covered by the HE-IRS. We distinguish the location of the user in that area by the corresponding angle of departure (AoD) of the LoS path of the IRS–User channel, thus establishing an angular space for the user in this area. Specifically, the azimuth AoD $\theta_{t}\in [\theta_{t}^{min},\;\theta_{t}^{max}]$ and the elevation AoD $\zeta_{t}\in [\zeta_{t}^{min},\zeta_{t}^{max}]$, where $\theta_{t}^{min}(\theta_{t}^{max})$ and $\zeta_{t}^{min}(\zeta_{t}^{max})$ are the minimum (maximum) values of the azimuth and elevation AoDs of this angular space. Then, we randomly select $N_{1}$ and $N_{2}$ grids within $\left[\theta_{t}^{min},\;\theta_{t}^{max}\right]$ and $\left[\zeta_{t}^{min},\zeta_{t}^{max}\right]$, thereby establishing $N=N_{1}N_{2}$ angular directions of the IRS–User channel. Therefore, the BS–IRS channel can be represented by $h={\left[h_{1},..,h_{M}\right]}^{T}\in C^{M\times 1}$ while the IRS–User channel at the *n*-th (n=1,…,N) angular direction can be denoted as $g_{n}={\left[g_{n,1},..,g_{n,M}\right]}^{T}\in C^{M\times 1}$.

As we mentioned in Section 2.1, the HE-IRS consists of both DTEs and STEs. We define a sparsity factor τ as the ratio of the number of STEs to the total number of reflecting elements. Thus, the number of STEs can be represented as τM, while that of DTEs can be represented as (1−τ)M. To identify the positions of DTEs and STEs, we also define an element position vector $a≜{\left[a_{1},a_{2},\ldots ,\;a_{M}\right]}^{T}$, where $a_{m}\in \{0,\;1\}$ is defined to distinguish whether a reflecting element is a DTE ($a_{m}=1$) or an STE ($a_{m}=0$). Therefore, the received signal at the *n*-th user direction can be expressed as follows:

(1) $$\begin{array}{c} y_{n}=\sum_{m=1}^{M}\left(1-a_{m}\right)g_{n,m}^{*}\phi_{m}h_{m}s_{n}+\sum_{m=1}^{M}a_{m}g_{n,m}^{*}\varphi_{n,m}h_{m}s_{n}+z_{n}, \end{array}$$

where $\phi_{m}$ denotes the phase shift at the *m*-th DTE and $\varphi_{n,m}$ represents the phase shift at the *m*-th STE associated with the *n*-th angular direction. $s_{n}$ is the BS-transmitted symbol intended to the user located at the *n*-th direction of the HE-IRS-covering area. $z_{n}\sim CN(0,\sigma^{2})$ denotes the additive white Gaussian noise with covariance $\sigma^{2}$ at that user. It can be shown that (1) can be further rewritten as follows:

(2) $$\begin{array}{c} y_{n}=g_{n}^{H}\left(\tilde{A}⊙\Phi \right)hs_{n}+g_{n}^{H}\left(A⊙\Psi_{n}\right)hs_{n}+z_{n}, \end{array}$$

where $A=diag(a)\in C^{M\times M}$ is a diagonal matrix with the element position vector a on its main diagonal, and $\tilde{A}$ is implemented by $I_{M}-A$. $\Phi =diag(\phi )\in C^{M\times M}$ with $\phi ={\left[\phi_{1},\phi_{2},\ldots ,\phi_{M}\right]}^{T}\in C^{M\times 1}$ and $\Psi_{n}=diag(\varphi_{n})\in C^{M\times M}$ with $\varphi_{n}={\left[\varphi_{n,1},\ldots ,\varphi_{n,M}\right]}^{T}\in C^{M\times 1}$ represent the diagonalized phase shift matrices of the DTEs and the STEs, respectively.

The BS–IRS channel is modeled as an LoS-dominated channel, expressed as follows:

(3) $$\begin{array}{c} h=\alpha a\left(\theta_{r},\zeta_{r}\right), \end{array}$$

where α represents the path loss component and $a\left(\theta_{r},\zeta_{r}\right)$ is the array response vector parameterized by the azimuth AoA $\theta_{r}$ and elevation AoA $\zeta_{r}$ from the BS.

Similarly, the IRS–User channel is also modeled as an LoS-dominated channel, given by the following:

(4) $$g_{n}=\beta a\left(\theta_{t},\zeta_{t}\right),$$

where β represents the path loss, and $a\left(\theta_{t},\zeta_{t}\right)$ is the array response vector determined by the user’s azimuth AoD $\theta_{t}$ and elevation AoD $\zeta_{t}$ from the HE-IRS. For the uniform planar array (UPA) in the *yz*-plane with $M_{y}$ and $M_{z}$ elements on the *y* and *z* axes, respectively, its specific form is given as follows:

(5) $$\left\{\begin{array}{c} \begin{array}{c} a\left(\theta_{r},\zeta_{r}\right)=a_{y}\left(\theta_{r},\zeta_{r}\right)⊗a_{z}\left(\zeta_{r}\right)\\ a\left(\theta_{t},\zeta_{t}\right)=a_{y}\left(\theta_{t},\zeta_{t}\right)⊗a_{z}\left(\zeta_{t}\right) \end{array}\\ f\left(u,N\right)=\frac{1}{\sqrt{M}}{\left[1,e^{j\pi u},\ldots ,e^{j\pi \left(N-1\right)u}\right]}^{T}\\ a_{y}=f\left(\sin\theta_{r\left(t\right)}\sin\zeta_{r\left(t\right)},M_{y}\right),a_{z}=f\left(\cos\zeta_{r\left(t\right)},M_{z}\right). \end{array}\right.$$

### 2.3. Problem Formulation

Given that the user can be located in any position within the coverage area of the HE-IRS, all potential angular directions of the user should be considered. In this section, we formulate the optimization problem of the HE-IRS-aided mmWave communication system, where the element position vector a, the phase shift vector of the STEs ϕ, and the phase shift vector of the DTEs $\varphi_{n}$ are jointly designed, to maximize the average received power over all possible user directions of this angular space.

For the convenience of subsequent optimization, we can further reform (2) as follows:

(6) $$\begin{array}{c} y_{n}={\left(g_{n}⊙h^{*}\right)}^{H}\left(\tilde{a}⊙\phi \right)s_{n}+{\left(g_{n}⊙h^{*}\right)}^{H}\left(a⊙\varphi_{n}\right)s_{n}+z_{n}, \end{array}$$

where $\tilde{a}$ is defined as $1_{N\times 1}-a$. Therefore, the received power of the system at the *n*-th direction can be expressed as follows:

(7) $$P_{n}={\left\|{\left(g_{n}⊙h^{*}\right)}^{H}\left(\tilde{a}⊙\phi \right)+{\left(g_{n}⊙h^{*}\right)}^{H}\left(a⊙\varphi_{n}\right)\right\|}^{2}.$$

Without a loss of generality, we assume that the transmit power $P_{n}$ is equivalent for all angular directions.

Therefore, we aim to maximize the average received power over all angular directions throughout this angular space by jointly optimizing the element position and the phase shifts of the HE-IRS. The optimization problem is formulated as follows:

(8) $$\begin{array}{c} \begin{array}{cc} \underset{a,\phi ,\varphi_{n}}{\mathrm{maximize}} & \frac{1}{N}\sum_{n=1}^{N}P_{n}\\ \mathrm{subject}\;\mathrm{to} & a_{m}\in \left\{0,1\right\},\forall m,\\ & {\left|a\right|}_{0}=\left(1-\tau \right)M,\\ & \left|\phi_{m}\right|=1,\;\forall m,\\ & \varphi_{n,m}\in F,\;\forall n,m. \end{array} \end{array}$$

The third and fourth constraints are due to the finite- and infinite-precision phase shifts enabled by the DTEs and the STEs, as mentioned in Section 2.1, where $F=\{1,e^{j\frac{2\pi }{L}},\ldots ,e^{j\frac{2\pi (L-1)}{L}}\},$ and $L=2^{b}$ is the set of discrete phase shift values at each DTE with *b* bits. Equation (8) demonstrates that DTEs and STEs each provide unique benefits: DTEs afford a greater design flexibility, while STEs offer an enhanced phase-shift resolution, and the HE-IRS capitalizes on the strategic positions of these two types of reflecting elements to achieve a better balance.

## 3. Element Position and Phase Shift Optimization

Due to the multiple coupled optimization variables and the highly nonconvex 0–1 constraints, it is difficult to directly solve problem (8). In this section, we decouple the element position and phase shift optimization of the reflecting elements, and firstly solve the position optimization problem with fixed phase shifts for all elements. Then, we decompose the phase shift optimization of the DTEs and STEs and alternatively optimize the phase shifts of the two types of reflecting elements.

### 3.1. Element Position Optimization

For the initialization of the phase shifts $\varphi_{n}$, we assume that all positions on the HE-IRS are occupied by DTEs, which means that the HE-IRS can be treated similarly to a conventional IRS. Then, we can obtain a closed-form solution for the phase shifts of all reflecting elements with the channel information known in the SISO scenario [13]. We take this solution as the initial phase shifts for $\varphi_{n}$. Then, the optimization problem (8) can be formulated as follows:

(9) $$\begin{array}{cc} \underset{a}{\mathrm{maximize}} & \frac{1}{N}\sum_{n=1}^{N}{\left\|{\left(g_{n}⊙h^{*}\right)}^{H}\left(a⊙\varphi_{n}\right)\right\|}^{2}\\ \mathrm{subject}\;\mathrm{to} & a_{m}\in \left\{0,1\right\},\forall m,\\ & {\left\|a\right\|}_{0}=\left(1-\tau \right)M. \end{array}$$

We incorporate the $l_{0}$-norm constraint of the element position vector a as a penalty term into the objective function, which is formulated as follows:

(10) $$\begin{array}{c} \begin{array}{cc} \underset{a}{\mathrm{maximize}} & \frac{1}{N}\sum_{n=1}^{N}{\left\|{\left(g_{n}⊙h^{*}\right)}^{H}\left(a⊙\varphi_{n}\right)\right\|}^{2}-\beta \xi \\ \mathrm{subject}\;\mathrm{to} & a_{m}\in \left\{0,1\right\},\forall m, \end{array} \end{array}$$

where β is the penalty coefficient, and the penalty function ξ can be expressed in the following form:

(11) $$\xi =\left\{\begin{array}{l} \left({\left\|a\right\|}_{0}-\tau M\right),\;\;{\;\;\;\;\;\;\;\;\;\;\;\left\|a\right\|}_{0}>M,\\ \;\;\;\;\;\;\;\;\;0{\;\;\;\;\;\;\;\;\;\;,\;\;\;\;\;\;\;\;\;\;\;\;\;\;\left\|a\right\|}_{0}<M. \end{array}\right.$$

It turns out that (10) is still difficult to solve due to the 0–1 constraint of a. Here, we adopt the PSO algorithm to achieve a local optimum of this complex problem.

The core of the PSO algorithm lies in the update rules of particle velocities and locations. The basic idea is to enable particles to gradually approach the global optimum in the process of searching for the optimal solution in the problem space by constantly communicating and cooperating. The entire execution process of the algorithm is similar to a collaborative search process of swarm intelligence.

Based on the basic principles of the PSO algorithm, the optimization of element position can be summarized as follows:

(1) Initialize the particle swarm: Randomly generate a certain number of particles, each of which has randomly chosen location and velocity, e.g., $x_{i}=\left(x_{i1},\;x_{i2},\;\ldots ,x_{iM}\right)$ and $v_{i}=\left(v_{i1},\;v_{i2},\;\ldots ,v_{iM}\right)$ for the *i*-th particle. In particular, the location vector $x_{i}$ represents the element position vector a in (10), while the velocity vector $v_{i}$ represents the changing trend of $x_{i}$ during iterations.
(2) Compute the fitness function: For each particle, compute the value of the fitness, which measures the quality of the solution in the problem space. The fitness function includes the local optimum of a particle and the global optimum of all particles, e.g., $p_{i,best}=\left(p_{i1},\;p_{i2},\;\ldots ,p_{iM}\right)$ and $g_{best}=\left(g_{i1},\;g_{i2},\;\ldots ,g_{iM}\right)$ for the *i*-th particle.
(3) Update the particle velocity and location: Update the velocity and location of each particle based on its current velocity, location, local optimum, and global optimum in order to move towards a better solution. That is,

(12) $$\begin{array}{c} x_{ij}\left(t+1\right)=x_{ij}\left(t\right)+v_{ij}\left(t+1\right), \end{array}$$

(13) $$v_{ij}\left(t+1\right)=w\cdot v_{ij}\left(t\right)+c_{1}r_{1}\left(t\right)\left[p_{ij}\left(t\right)-x_{ij}\left(t\right)\right]+c_{2}r_{2}\left(t\right)\left[g_{j}\left(t\right)-x_{ij}\left(t\right)\right],$$

(14) $$w=w_{max}-\frac{\left(w_{max}-w_{min}\right)t}{T_{max}},$$

where $c_{k}$ is the learning factor, while $r_{k}$ is a random number within [0, 1]. $v_{ij}\in [-v_{max},\;v_{max}]$ is the velocity of particle, with $v_{max}$ denoting the maximum velocity.

However, since the element position vector $x_{i}$ is a discrete variable, we need to employ a discrete PSO algorithm. Therefore, we define the discretization rules as follows:

(15) $$s\left(v_{i,j}\right)=\frac{1}{\left[1+\exp\left(-v_{i,j}\right)\right]},$$

(16) $$x_{i,j}=\left\{\begin{array}{l} 1,r<s\left(v_{i,j}\right)\\ 0,\;\mathrm{others}\; \end{array},\right.$$

where $s\left(v_{i,j}\right)$ is a threshold that determines whether the element position variable $x_{i,j}$ takes 0 or 1.

(4) Check the stopping criterion: If the specified stopping criterion is met, such as reaching the required number of iterations or finding a satisfactory solution, the algorithm stops and returns the optimal solution. Otherwise, the algorithm returns to step 2 and continues iterating.

### 3.2. The PSO-MO Algorithm for Phase Shift Optimization

After the element position optimization, the joint optimization of the phase shifts of the HE-IRS remains challenging due to their non-convex constraints and highly coupled nature. Therefore, we decouple the phase shifts of the DTEs and STEs and alternately optimize them. The detailed process of the phase shift optimization can be expressed as follows:

(1) Initializing the phase shifts of the DTEs

Firstly, we initialize the phase shifts of the DTEs, i.e., $\varphi_{n}$, for all angular directions. In this case, we focus on the contribution of the DTEs, and then the original optimization problem (9) can be rewritten as follows:

(17) $$\begin{array}{cc} \underset{{\tilde{\varphi }}_{n}}{\mathrm{maximize}} & \frac{1}{N}\sum_{n=1}^{N}{\left\|{\left({\tilde{g}}_{n}⊙{\tilde{h}}^{*}\right)}^{H}{\tilde{\varphi }}_{n}\right\|}^{2}\\ \mathrm{subject}\;\mathrm{to} & {\tilde{\varphi }}_{n,m}\in F,\;\forall n,m, \end{array}$$

where $\tilde{h}\in C^{(1-\tau )M\times 1}$ and ${\tilde{g}}_{n}\in C^{(1-\tau )M\times 1}$ are the BS–IRS channel and the IRS–User channel corresponding to the DTEs, respectively. ${\tilde{\varphi }}_{n}\in C^{(1-\tau )M\times 1}$ represents the phase shift vector of the DTEs. To maximize the objective function in (17), which is to maximize the received power at every angle, it is crucial to consider the multiplication of a complex row vector with a complex column vector. The product of these vectors reaches its maximum when one is the conjugate transpose of the other. From this principle, a closed-form solution can be deduced, offering an expression with infinite precision, as follows:

(18) $${\tilde{\varphi }}_{n}=e^{-j\left(\arg\left({\tilde{g}}_{n}⊙{\tilde{h}}^{*}\right)+2l\pi \right)},l=0,\pm 1,\ldots .$$

Due to the finite-precision phase shifts of the DTEs, the actual phase can be represented as ${\tilde{\varphi }}_{n,m}^{ini}=$ $e^{j\frac{k^{*}\pi }{2^{L-1}}}$, where $k^{*}=\underset{k}{\mathrm{argmin}}\left|e^{j\frac{k\pi }{2^{L-1}}}-{\tilde{\varphi }}_{n,m}\right|,k=0,1,\ldots ,L-1$.

(2) Optimizing the phase shifts of the STEs

Given a fixed elements position vector a and a fixed phase shift vector of the DTEs ${\tilde{\varphi }}_{n}$, the optimization problem (9) is only associated with the phase shift vector of the STEs $\tilde{\phi }\in C^{\tau M\times 1}$, which can be expressed as follows:

(19) $$\begin{array}{cc} \underset{\bar{\phi }}{\mathrm{maximize}} & \frac{1}{N}\sum_{n=1}^{N}{\left\|{\left({\bar{g}}_{n}⊙{\bar{h}}^{*}\right)}^{H}\bar{\phi }+{\left({\tilde{g}}_{n}⊙{\tilde{h}}^{*}\right)}^{H}{\tilde{\varphi }}_{n}\right\|}^{2}\\ \mathrm{subject}\;\mathrm{to} & \left|{\bar{\phi }}_{m}\right|=1,\;\forall m, \end{array}$$

where $\bar{h}\in C^{\tau M\times 1}$ and ${\bar{g}}_{n}\in C^{\tau M\times 1}$ are the BS–IRS channel and the IRS–User channel corresponding to the STEs, respectively. $\bar{\phi }\in C^{\tau M\times 1}$ represents the phase shift vector of the STEs.

To deal with the non-convex constant modulus constraint, we adopt the MO method to find a locally optimal solution for this problem [28]. The basic principle of MO is to treat the feasible set as a manifold. The key idea is to optimize the objective function on the manifold by following a path that stays on the manifold at each iteration. This is achieved by using Riemannian geometry to define a distance metric on the manifold, and then performing optimization using gradient-based methods that respect the manifold structure. Thus, MO can efficiently explore the feasible set and find high-quality solutions to challenging optimization problems.

**Figure 3:**
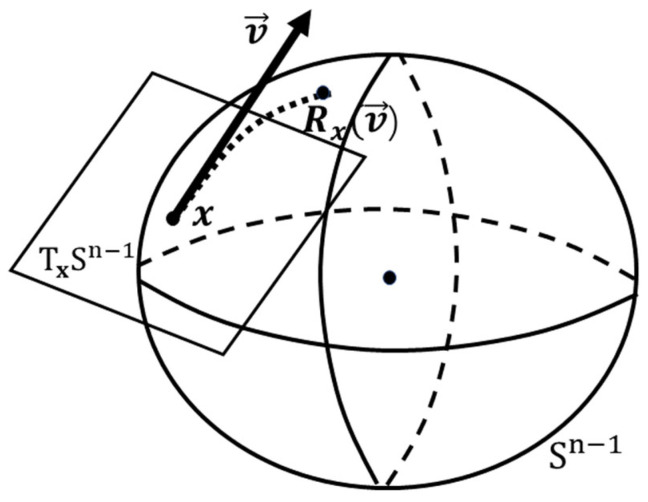
The tangent space and tangent vector of a Riemannian manifold.

As shown in Figure 3, the tangent space $T_{x}S^{n-1}$ and tangent vector v are on the complex circle manifold $S^{n-1}$ at a given point x. The tangent space $T_{x}S^{n-1}$ is spanned by the tangent vectors at a given point. During complex circle manifold optimization, we need to project the gradient onto $T_{x}S^{n-1}$ to ensure that the optimization direction stays within the tangent space and does not leave the circle. The tangent vector v is used to calculate the distances and angles between tangent vectors, which are necessary for determining the optimization direction and step size. These operations are defined using the inner product between the tangent vectors and the metric on the tangent space $T_{x}S^{n-1}$, which follows the rules of non-Euclidean geometry.

Among all the tangent vectors, one of them that is related to the negative Riemannian gradient represents the direction of the greatest decrease. As the complex circle manifold $S^{n-1}$ is a Riemannian submanifold of $C^{m}$, the Riemannian gradient at x is a tangent vector gradf(x) given by the orthogonal projection of the Euclidean gradient ∇f(x) onto the tangent space $T_{x}S^{n-1}$, as follows:

(20) $$\begin{array}{c} gradf(x)={Proj}_{x}\nabla f(x)\\ \begin{array}{c} =\nabla f(x)-R\left\{\nabla f(x)⊙x^{*}\right\}⊙x, \end{array} \end{array}$$

where the Euclidean gradient of the cost function is as follows:

(21) $$\nabla f\left(x\right)=-\left(\sum_{n=1}^{N}{\left({\bar{g}}_{n}⊙{\bar{h}}^{*}\right)\left({\bar{g}}_{n}⊙{\bar{h}}^{*}\right)}^{H}\right)\bar{\phi }-\sum_{n=1}^{N}\left({\tilde{g}}_{n}⊙{\tilde{h}}^{*}\right){\left({\tilde{g}}_{n}⊙{\tilde{h}}^{*}\right)}^{H}{\tilde{\varphi }}_{n}.$$

The Euclidean gradient is obtained by taking the conjugate gradient of the loss function, which involves some techniques on complex-valued matrix derivatives.

In MO, retraction is a key concept that is used to map the tangent vectors back to the manifold after performing optimization operations. The retraction maps a tangent vector to a new point on the manifold so that the optimization process stays on the manifold. The retraction is defined using a smooth map that maps a tangent vector at a given point to a new point on the manifold. The smoothness property ensures that the retraction preserves the local geometry of the manifold and avoids introducing singularities or discontinuities. The retraction of a tangent vector ct at point $x\in S^{n-1}$ can be stated as follows:

(22) $$\begin{array}{c} {Retraction}_{x}:T_{x}S^{n-1}\to S^{n-1}, \end{array}$$

(23) $$\begin{array}{c} ct\to {Retraction}_{x}\left(ct\right)=vec\left[\frac{{\left(x+ct\right)}_{k}}{\left|{\left(x+ct\right)}_{k}\right|}\right]. \end{array}$$

Based on the tangent space $T_{x}S^{n-1}$, Riemannian gradient gradf(x), and retraction on the complex circle manifold $S^{n-1}$, a linear search method can be utilized to design an adjustable infinite-precision phase, as shown in Algorithm 1.
**Algorithm 1** The optimization of the phase shifts of the STEs based on MO**Input:** 
${\bar{g}}_{n},\bar{h},{\tilde{g}}_{n},\tilde{h},a,{\tilde{\varphi }}_{n}^{ini}$
1: Initialize $\bar{\phi }$ with random phases, i=02: **repeat**    3:        Select the step size μ    4:        Update $vec\left(\bar{\phi }\right)$ according to (23)    5:        Update the Riemannian gradient $g_{i}=\nabla f\left(\bar{\phi }\right)$ according to (20)(21)    6:        Calculate $g_{i}^{+},d_{i}^{+}$ from $x_{i}$ to $x_{i+1}$    7:        Select Polak–Ribiere parameter $\eta_{i+1}$    8:        Calculate the conjugate direction $d_{i+1}=-g_{i+1}+\eta_{i+1}d_{i}^{+}$    9:            Update i←i+110: **until** a stopping condition is satisfied**Output:**  
$\bar{\phi }$

(3) Optimizing the phase shifts of the DTEs

Given a fixed position selection vector a and a fixed phase shift vector of the STEs $\bar{\phi }$, the optimization problem according to the phase shifts of the DTEs can be formulated as follows:

(24) $$\begin{array}{cc} \underset{{\tilde{\varphi }}_{n}}{\mathrm{maximize}} & \frac{1}{N}\sum_{n=1}^{N}{\left\|{\left({\bar{g}}_{n}⊙{\bar{h}}^{*}\right)}^{H}\bar{\phi }+{\left({\tilde{g}}_{n}⊙{\tilde{h}}^{*}\right)}^{H}{\tilde{\varphi }}_{n}\right\|}^{2}\\ \mathrm{subject}\;\mathrm{to} & {\tilde{\varphi }}_{n,m}\in F,\;\forall n,m. \end{array}$$

Similar to obtaining the closed-form solution (18), to maximize the objective function in (24), it is necessary to maximize the received power at every angle. Given that the product of two complex vectors, a row vector and a column vector, is maximized when they are conjugate transposes of each other, we can derive that for DTEs with infinite precision, the closed-form solution is as follows:

(25) $${\tilde{\varphi }}_{n}^{opt}=e^{j\left(\arg\left({\left({\bar{g}}_{n}⊙{\bar{h}}^{*}\right)}^{H}\bar{\phi }\right)-\arg\left({\tilde{g}}_{n}⊙{\tilde{h}}^{*}\right)+2l\pi \right)},l=0,\pm 1,\ldots ,$$

similar to the initialization of the DTEs, the actual phase shift vector can be represented as ${\tilde{\varphi }}_{n,m}^{opt}=e^{j\frac{k^{*}\pi }{2^{L-1}}}$, where $k^{*}=\underset{k}{\mathrm{argmin}}\left|e^{j\frac{k\pi }{2^{L-1}}}-{\tilde{\varphi }}_{n,m}\right|,k=0,1,\ldots ,L-1$.

We develop a joint element position and phase shift design algorithm iteratively and alternatively. During each iteration, with the fixed ${\tilde{\varphi }}_{n}$ and $\bar{\phi }$, we first optimize the position selection vector a according to PSO. Then, fixing the element position vector a, we optimize ${\tilde{\varphi }}_{n}$ and $\bar{\phi }$ alternatively. Specifically, we optimize the phase shifts of the STEs by Algorithm 1, and then obtain the closed-form solution for the phase shifts of the DTEs by fixing the STEs phase shifts. These steps are repeated until the stopping condition is satisfied. The overall optimization algorithm is summarized in Algorithm 2, which is referred to as the PSO-MO algorithm.
**Algorithm 2** The PSO-MO algorithm**Input:**${\bar{g}}_{n},\bar{h},{\tilde{g}}_{n},\tilde{h}$1:  Initialize $\bar{\phi },{\tilde{\varphi }}_{n},\;i=0$2:  Optimize a by solving problem (10) via the PSO algorithm3:    **repeat:**4:                 Initialize ${\tilde{\varphi }}_{n}$ according to (18)5:                 Compute $\bar{\phi }$ according to Algorithm 16:                 Compute ${\tilde{\varphi }}_{n}$ according to (25)7:                 i←i+18:    **until** a stopping condition is satisfied **Output:**  a, $\bar{\phi },$ ${\tilde{\varphi }}_{n}$

### 3.3. The PSO-CSB Algorithm for Phase Shift Optimization

After the element position vector fixed by the PSO, in this section, we propose a method for optimizing the phase shifts of both the DTEs and STEs with a relatively low complexity. Specifically, we use the Cauchy–Schwarz inequality to transform the original objective function into its lower bound and obtain closed-form solutions for the phase shifts of the DTEs and STEs. Specifically, the Cauchy–Schwarz inequality can be written as follows:

(26) $$\left(\sum_{k=1}^{K}a_{k}^{2}\right)\left(\sum_{k=1}^{K}b_{k}^{2}\right)\geq {\left(\sum_{k=1}^{K}a_{k}b_{k}\right)}^{2}.$$

Using (26), the original objective function can be transformed into the following inequality:

(27) $$\begin{array}{c} \sum_{n=1}^{N}{\left\|{\left({\bar{g}}_{n}⊙{\bar{h}}^{*}\right)}^{H}\bar{\phi }+{\left({\tilde{g}}_{n}⊙{\tilde{h}}^{*}\right)}^{H}{\tilde{\varphi }}_{n}\right\|}^{2}\\ \geq {\left\|\sum_{n=1}^{N}\left({\left({\bar{g}}_{n}⊙{\bar{h}}^{*}\right)}^{H}\bar{\phi }+{\left({\tilde{g}}_{n}⊙{\tilde{h}}^{*}\right)}^{H}{\tilde{\varphi }}_{n}\right)\right\|}^{2}\\ ={\left\|{\left(\sum_{n=1}^{N}\left({\bar{g}}_{n}⊙{\bar{h}}^{*}\right)\right)}^{H}\bar{\phi }+\sum_{n=1}^{N}\left({\left({\tilde{g}}_{n}⊙{\tilde{h}}^{*}\right)}^{H}{\tilde{\varphi }}_{n}\right)\right\|}^{2}. \end{array}$$

Therefore, the maximization of the objective function in the original problem can be transformed into the maximization of its lower bound, i.e., (27). Fortunately, both the phase shift vector of the DTEs ${\tilde{\varphi }}_{n}$ and the phase shift vector of the STEs $\bar{\phi }$ have closed-form solutions, which can be given as follows:

(28) $${\bar{\phi }}^{low}=e^{j\left(\arg\left(\sum_{n=1}^{N}\left({\left({\tilde{g}}_{n}⊙{\tilde{h}}^{*}\right)}^{H}{\tilde{\varphi }}_{n}\right)\right)-\arg\left({\bar{g}}_{n}⊙{\bar{h}}^{*}\right)+2l_{1}\pi \right)},l_{1}=0,\pm 1,\ldots ,$$

(29) $${\tilde{\varphi }}_{n}=e^{-j\left(\arg\left({\tilde{g}}_{n}⊙{\tilde{h}}^{*}\right)+2l_{2}\pi \right)},l_{2}=0,\pm 1,\ldots .$$

Due to the finite-precision phase shifts of the DTEs, the actual phase shift vector can be represented as ${\tilde{\varphi }}_{n,m}^{low}=e^{j\frac{k^{*}\pi }{2^{L-1}}}$, where $k^{*}=\underset{k}{\mathrm{argmin}}\left|e^{j\frac{k\pi }{2^{L-1}}}-{\tilde{\varphi }}_{n,m}\right|,k=0,1,\ldots ,L-1$.

By using (28) and (29), we can obtain the solutions of $\bar{\phi }$ and ${\tilde{\varphi }}_{n}$, and then take them into (27) to derive the Cauchy–Schwarz bound. The optimization algorithm for the element position and phase shifts of the DTEs and STEs is summarized in Algorithm 3, which is referred to as the PSO-CSB algorithm.
**Algorithm 3** The PSO-CSB algorithm**Input:  **${\bar{g}}_{n},\bar{h},{\tilde{g}}_{n},\tilde{h}$1:  Initialize $a,\bar{\phi },{\tilde{\varphi }}_{n},\;i=0$2:  Compute a according to (10)3:  Compute $\bar{\phi }$ according to (28)4:  Compute ${\tilde{\varphi }}_{n}$ according to (29)**Output** 
**:  **
$a,\bar{\phi },{\tilde{\varphi }}_{n}$


It is worth noting that Algorithm 3 does not require any iteration, which implies the low complexity of the proposed PSO-CSB algorithm. However, since the original problem is relaxed by using the Cauchy–Schwarz inequality, the beamforming solution obtained by this method is only equivalent to the solution of the original problem when the lower bound is tight. Therefore, due to the relaxation, the performance of this method is often not as good as PSO-MO, as will be shown in the next section.

## 4. System Evaluation

In this section, we first discuss the convergence of all the proposed algorithms, followed by an analysis of their computational complexity.

### 4.1. Convergence

It is noteworthy that both the PSO-MO and the PSO-CSB algorithms utilize the PSO algorithm for the optimization of element position. Thus, we first prove the convergence of the PSO algorithm. For the PSO-MO algorithm, the presence of a closed-form solution for the phase shifts of DTEs ensures a consistent increase in the objective function. Subsequently, within this algorithm, the convergence of the MO algorithm employed for the STEs will be demonstrated. As for the PSO-CSB algorithm, the convergence of the CSB algorithm will also be subsequently proven.

(1) PSO: In this algorithm, particles have memory, implying that throughout the search process, the local optimum for any particle at any given moment may evolve. Thus, the global optimum across all particles will be guaranteed to remain non-decreasing. This characteristic ensures that the search process is directed towards potential optima, and this is fundamental to the convergence properties of the algorithm.
(2) MO: The phase shifts of the STEs are optimized utilizing the MO method. In light of Theorem 4.3.1 presented in [29], it is guaranteed that the algorithm employing the MO method will converge to a point where the gradient of the objective function gradient vanishes. Consequently, each iteration within the overarching alternating algorithm for phase shift optimization is assured to enhance the value of the objective function, thus allowing for a rigorous proof of convergence.
(3) CSB: In this algorithm, convergence is also ensured, since a closed-form solution is obtained directly through the application of the Cauchy–Schwarz inequality, eliminating the need for an iterative process.

In summary, given the fact that the PSO, MO, and CSB algorithms each maintain convergence properties, it follows that both the PSO-MO and PSO-CSB algorithms are convergent.

### 4.2. Complexity Analysis

In this subsection, we analyze computational complexity in terms of the number of complex multiplications for both the proposed algorithms.

For the PSO-MO algorithm, the main complexity includes the following:

(1) The PSO algorithm: According to the fitness function described by Equation (10), the computational complexity of the fitness function is *2MN*. Then, the computational complexity of the PSO algorithm is approximately proportional to the product of the number of particles $N_{p}$, the number of iterations $I_{p}$, and the complexity of the fitness function *2MN* [30]. Therefore, it can be expressed as $N_{p}I_{p}2MN$.
(2) The MO algorithm for the STEs: The complexity of the MO algorithm can be divided into two main parts. Computation of the conjugate gradient: According to Equation (21), the total complexity in computing the gradient is $(M+\tau^{2}M^{2}+2{\left(1-\tau \right)}^{2}M^{2})N+\tau^{2}M^{2}$. Orthogonal projection and retraction operations: The orthogonal projection is essentially the Hadamard production, which takes 2τMN, and the complexity of the retraction operation is τMN. Therefore, the computational complexity of this algorithm can be described as $I_{m}((\tau^{2}M^{2}+2{\left(1-\tau \right)}^{2}M^{2}+(1+3\tau )M)N+\tau^{2}M^{2})$, where $I_{m}$ denotes the inner iterations for the MO algorithm.
(3) The closed-form solution for the DTEs: Based on Equation (25), the computational complexity of obtaining the closed-form solution is (τ+1)MN.

We denote the numbers of the iteration for the phase shift optimization of DTEs and STEs as $I_{phase}$, and the total complexity of the PSO-MO algorithm is $N_{p}I_{p}2MN+I_{phase}(I_{m}((\tau^{2}M^{2}+2{\left(1-\tau \right)}^{2}M^{2}+(3\tau +1)M)N+\tau^{2}M^{2})+(\tau +1)MN)$.

For the PSO-CSB algorithm, its complexity includes the following two parts: the PSO algorithm and the CSB algorithm. The complexity of the PSO algorithm has already been computed as $N_{p}I_{p}2MN$. For the complexity of the CSB algorithm, based on Equations (28) and (29), the computational complexities for calculating the phase shifts of the STEs and the DTEs are 2(1−τ)MN+τM and (1−τ)MN, respectively. Therefore, the total complexity of the PSO-CSB algorithm is $N_{p}I_{p}2MN+3(1-\tau )MN+\tau M$.

## 5. Simulation Results

In this section, we provide various numerical simulation results to evaluate the system performance assisted by the HE-IRS, which is optimized by the proposed PSO-MO and PSO-CSB algorithms, as well as the conventional IRS optimized by the MO algorithm. In order to observe the performance difference between the HE-IRS and the conventional IRS, we employ the received power ratio as a metric to quantify the impact imparted on the communication system by the HE-IRS relative to the conventional IRS, which can be expressed as follows:

(30) $$\eta =\frac{P_{HE-IRS}}{P_{IRS}},$$

where $P_{HE-IRS}$ and $P_{IRS}$ represent the received power of the communication system assisted by the HE-IRS and the conventional IRS, respectively.

We assume that the system is operating in an mmWave band with a carrier frequency of 28 GHz, and the LoS paths dominate in all channels, i.e., the BS–IRS channel and the IRS–User channel. The path loss for the BS–IRS and IRS–User links is calculated using: ${\left\|\alpha \right\|}^{2}=10^{-6.14-2.1{log}_{10}^{d_{BI}}},{\left\|\beta \right\|}^{2}=10^{-6.14-2.1{log}_{10}^{d_{IU}}}$, where the path loss is set to 61.4 dB at 1 m, with a path loss exponent of 2.1. The distance between the BS and the IRS is set to $d_{BI}=10$ m, and the distance between the IRS and the user is set to $d_{IU}=5$ m. The BS–IRS channel h is assumed to be fixed due to the stationary nature of the BS and IRS. For simulations, we set $\theta_{r}=30{}^{\circ}$ and $\zeta_{r}=60{}^{\circ}$. The IRS–User channel considers a user randomly located within a defined angular space. Specifically, $\theta_{t}$ and $\zeta_{t}$ are uniformly distributed in the ranges $\left[\theta_{t}^{min},\;\theta_{t}^{max}\right]$ and $\left[\zeta_{t}^{min},\;\zeta_{t}^{max}\right]$, respectively, with $\Delta \theta_{t}=\theta_{t}^{max}-\theta_{t}^{min}$ and $\Delta \zeta_{t}\;=\zeta_{t}^{max}-\zeta_{t}^{min}$. The simulation parameters are summarized in Table 1.

### 5.1. Convergence

**Figure 4:**
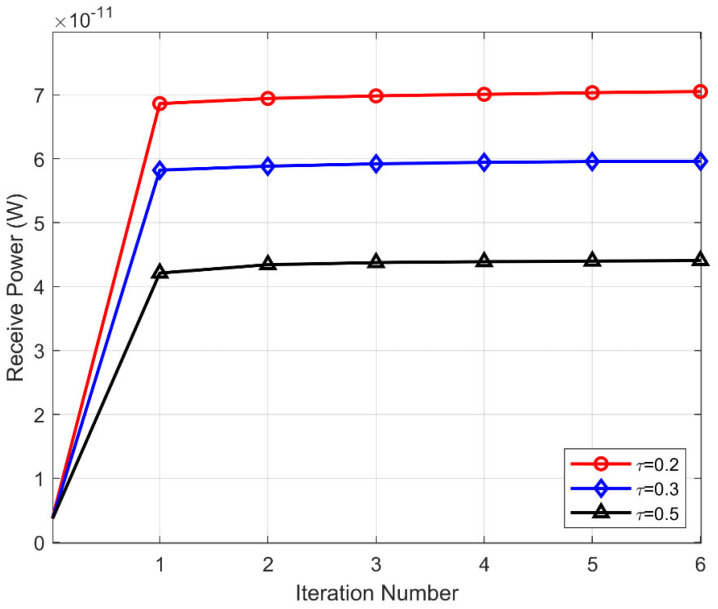
Convergence of the proposed PSOMO algorithm.

In Figure 4, we first show the convergence of the PSO-MO algorithm for different sparsity factors, with $\Delta \theta_{t}=60{}^{\circ},\Delta \zeta_{t}=120{}^{\circ},M=64,b=1\;bit$. It can be observed that the proposed PSO-MO algorithm quickly converges within a few iterations for all values of the sparsity factor τ. Meanwhile, by deploying more DTEs in HE-IRS, i.e., decreasing τ, the received power at the user can be significantly improved.

### 5.2. Received Power Ratio Versus the Angular Space

**Figure 5:**
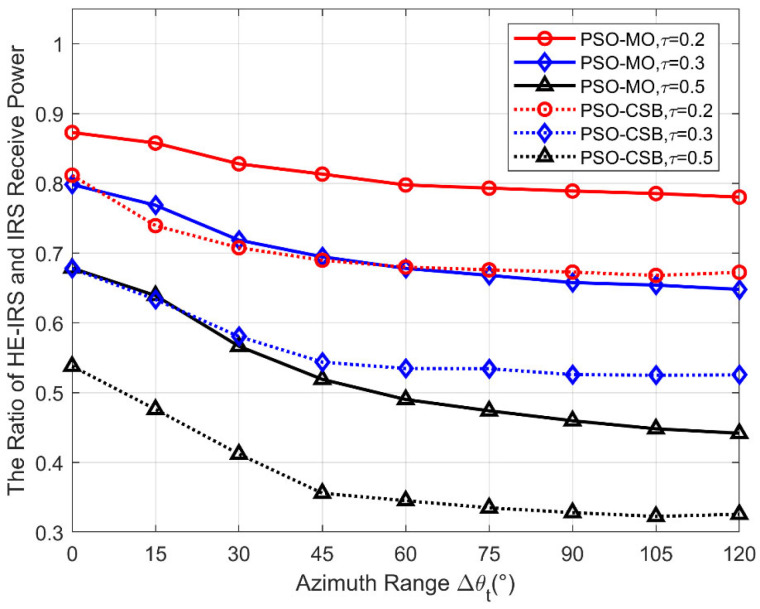
The ratio of received power versus azimuth range $\Delta \theta_{t}$.

In Figure 5, we evaluate the received power ratio of the proposed algorithms versus the size of the angular space, where we vary the range $\Delta \theta_{t}$ and fix $\Delta \zeta_{t}=120{}^{\circ},M=64,b=1\;bit$. As can be observed, the received power ratio of both the PSO-MO and PSO-CSB algorithms decreases gradually as the azimuth range $\Delta \theta_{t}$ increases for all τ. This is because as the STEs are fixed once the HE-IRS is produced, they can only focus the signal power to the whole potential angle range rather than specifically focusing on the user. Therefore, if the angle range extends, the signal power reflected by the STEs needs to be spread in a larger range, and the power at the specific angle where the user locates will be lower. It is also notable that an HE-IRS with fewer DTEs, i.e., larger τ, is much more sensitive to an increase in the azimuth angle range. For example, the performance of the τ = 0.2 HE-IRS designed by the PSO-MO algorithm decreases only from 87% to 78% when the azimuth angle range increases from 0° (fixed azimuth angle) to 120°. However, when τ = 0.5, the received power ratio decreases dramatically from 68% to 43%. Although the HE-IRS performs worse than the conventional IRS, the introduction of STEs can still bring a significant improvement in the received signal power, with lower power consumption and hardware costs. Based on the quadtratic relationship between the passive beamforming gain and the number of DTEs, as shown in [13], the performance of a sparse IRS (i.e., an IRS with only DTEs deployed at randomly selected positions, leaving the other positions void) can only achieve 64% and 25% for τ = 0.2 and τ = 0.5, respectively, which is significantly lower than the proposed HE-IRS with STEs. Finally, as the objective function of the PSO-CSB is not exactly the received power at the user but its lower bound, its performance is always worse than the PSO-MO algorithm. However, it still outperforms the sparse IRS with only DTEs for all τ, thanks to the beamforming effort of the STEs.

### 5.3. Received Power Ratio Versus the Number of Quantization Phase Shift Bits

**Figure 6:**
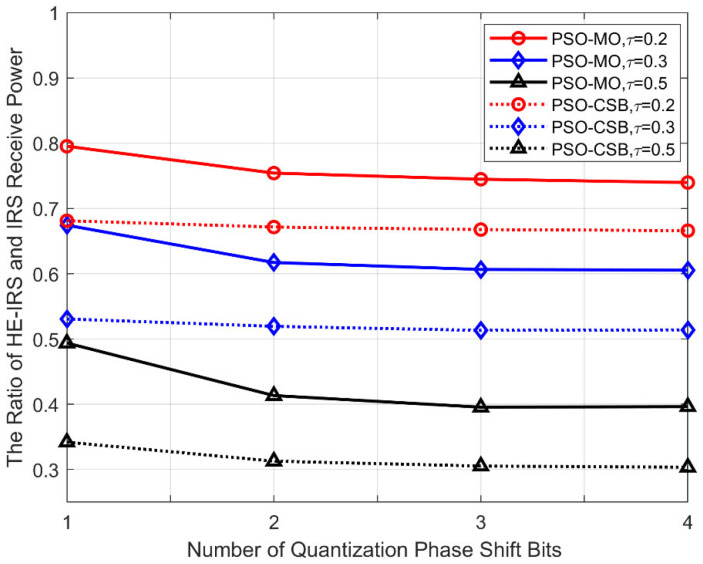
Received power ratio versus the number of quantization phase shift bits.

In Figure 6, we show the received power ratio of the proposed algorithms versus the number of quantization bits of the DTE phase shifts in the HE-RIS, with $\Delta \theta_{t}=60{}^{\circ},\;\Delta \zeta_{t}=120{}^{\circ},\;M=64$. As shown in this figure, the received power ratio of the PSO-MO and PSO-CSB algorithms decreases quickly when the number of quantization bits increases from one bit to two bits, but remains almost unchanged with a further increase in the number of quantization bits. This is because as the number of quantization bits increases, the quality of beamforming provided by them can be significantly improved. In this situation, as the conventional IRS has more DTEs compared to the HE-IRS, the benefits brought about by the infinite-precision phase shifts of the STEs are significantly weakened. Due to this reason, it can be observed that the influence of phase shift precision is relatively greater for higher sparsity levels.

### 5.4. Received Power Ratio Versus IRS Size

**Figure 7:**
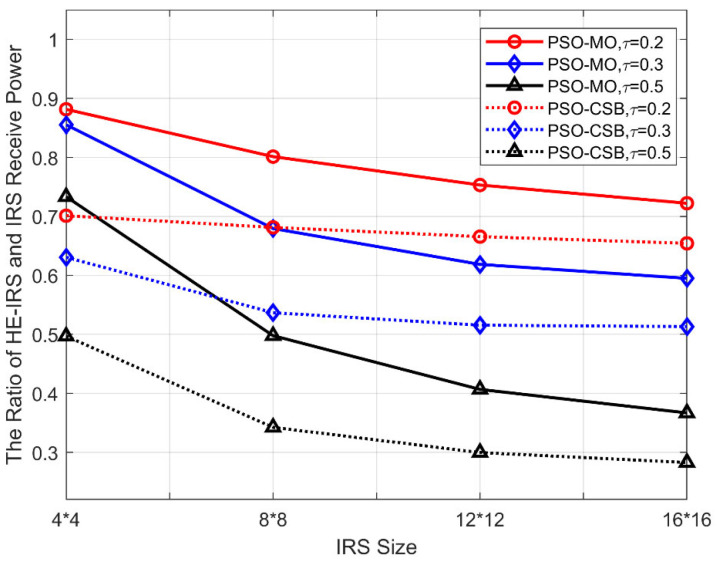
Received power ratio versus IRS size.

In Figure 7, we compare the received power ratio of the proposed algorithms versus the IRS size (the number of reflecting elements), with $\Delta \theta_{t}=60{}^{\circ},\Delta \zeta_{t}=120{}^{\circ},b=1\;bit$. As can be seen, the received power ratio of both algorithms decreases with the growth of the IRS. This is because with the increased size of the IRS and the fixed sparsity factor τ, the HE-RIS consists of more DTEs, and, thus, can significantly improve the beamforming quality. For the STEs, on the other hand, it cannot be reconfigured according to the varying channels during the beamforming, but can only focus the transmit signal to the given angular space. Due to this reason, the increase in the number of the STEs cannot significantly benefit the received power at the user. Consequently, with the increase in the IRS size, the performance of the conventional IRS can be improved at a much higher rate than the HE-IRS, thereby leading to a decreasing received power ratio. Due to a similar reason, the influence of the number of elements is greater for the HE-IRS with higher sparsity levels.

### 5.5. Received Power, Received Power Ratio, and Energy Efficiency Versus the Sparse Factor

**Figure 8:**
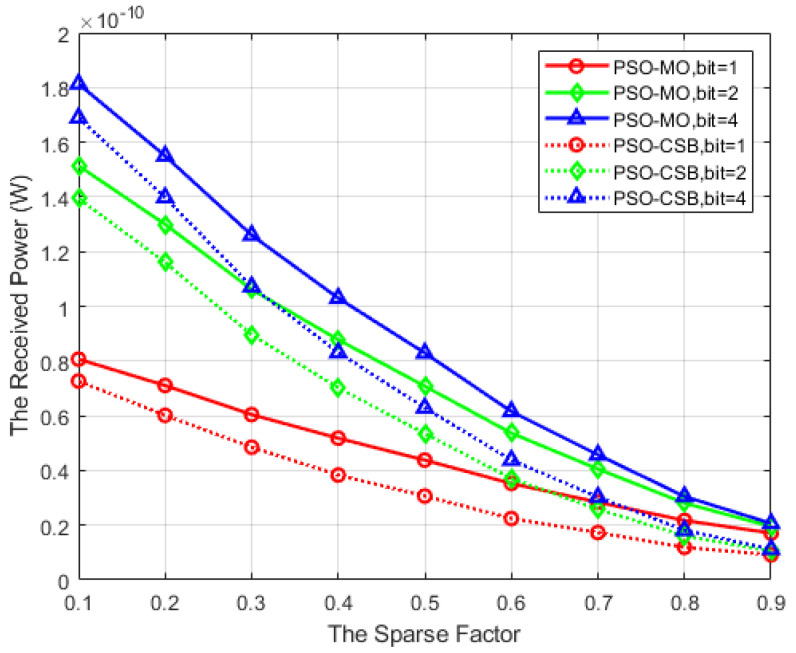
Received power versus the sparse factor.

In Figure 8, we evaluate the received power of the proposed algorithms versus the sparse factor τ in the HE-RIS, with $\Delta \theta_{t}=60{}^{\circ},\Delta \zeta_{t}=120{}^{\circ},M=64$. It can be observed from this figure that the received power for both the PSO-MO algorithm and the PSO-CSB algorithm decreases progressively as the sparse factor increases. This is attributed to the fact that with an increase in τ, there are more STEs involved, which leads to attenuation in the received power. However, it is also noteworthy that the power consumption of the entire IRS concurrently decreases. Simultaneously, the non-iterative PSO-CSB algorithm exhibits a performance closely aligned with the PSO-MO algorithm across all τ. As the number of quantization bits of the phase shifts in DTEs increases, their beamforming capability is enhanced, which, in turn, leads to a higher received power.

**Figure 9:**
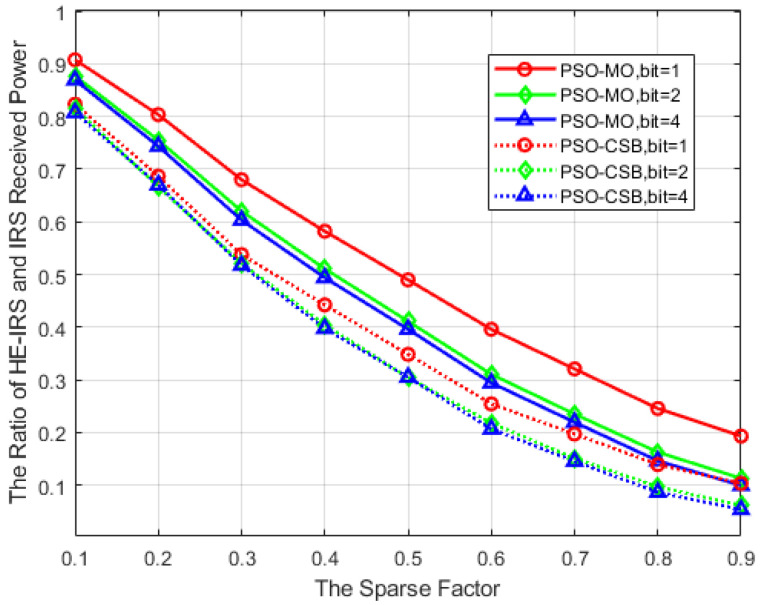
Received power ratio versus the sparse factor.

Furthermore, we plot the variation in the received power ratio against the sparse factor in Figure 9, with parameter settings consistent with those of Figure 8. It is observed that, unlike that in Figure 8, where an increase in the phase shift precision of the DTEs is associated with a higher received power, Figure 9 reveals that a greater phase shift precision leads to a reduction in the received power ratio. The underlying reasons for this phenomenon can be referred to in Part C of this section. However, the results depicted in this figure also suggest that the HE-IRS closely approximates the received power of the conventional IRS when the number of quantization bits of the phase shifts in DTEs is small.

**Figure 10:**
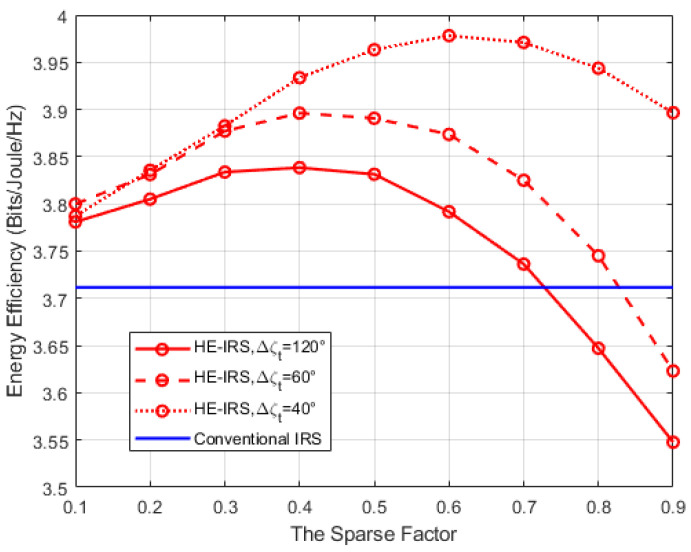
Energy efficiency versus the sparse factor.

Finally, Figure 10 shows the energy efficiency (EE) performance of the proposed HE-IRS system as a function of the sparse factor *τ* for different sizes of the user location area, when b=1 bit,M=64. Specifically, we fix the azimuth range at $\Delta \theta_{t}=60{}^{\circ}$, but change the elevation range $\Delta \zeta_{t}$. Similar to that in [8,31], the EE is defined as $R/P_{\mathrm{total}}$, where $R=\log(1+P/\sigma^{2})$ is the spectral efficiency (SE), with P denoting the received power at the user, and $P_{\mathrm{total}}=P_{b}+P_{\mathrm{cir}}+P_{\mathrm{ele}}$ is the total consumed power of a system, with $P_{b}$, $P_{\mathrm{cir}}=P_{\mathrm{cir}}^{\mathrm{BS}}+P_{\mathrm{cir}}^{\mathrm{UE}}$, and $P_{\mathrm{ele}}=MP_{\mathrm{DTE}}$ denoting the transmit power, the circuit power consumption of the BS ($P_{\mathrm{cir}}^{\mathrm{BS}}=30$ dBm) and the UE ($P_{\mathrm{cir}}^{\mathrm{UE}}=10$ dBm), and the reflecting element power consumed by the DTEs ($P_{\mathrm{DTE}}=10$ dBm), respectively. For comparison, we also provide the EE performance of the system assisted by the conventional IRS as a benchmark. As can be seen from the figure, the EE of the conventional IRS always remains unchanged, as all of its reflecting elements are DTEs. However, for the proposed HE-IRS architecture with the PSO-MO algorithm, the EE does not decrease monotonically, but initially increases before subsequently declining. This is because although the SE continuously decreases with an increase in τ, it is accompanied by a reduction in the power consumption $P_{\mathrm{ele}}$ of reflecting elements, as the STEs are entirely power-free. Furthermore, by comparing the three curves of the HE-IRS with different elevation ranges, it can be observed that the maximum EE values of these three curves are different and correspond to different values of the sparse factor. This is because the impact of STEs on the passive beamforming of the HE-IRS varies across different sizes of the user location area (i.e., the azimuth range or elevation range). Specifically, as the user location area narrows, the influence of the STEs on the whole passive beamforming becomes more significant.

### 5.6. Received Power Versus the Imperfect CSI

**Figure 11:**
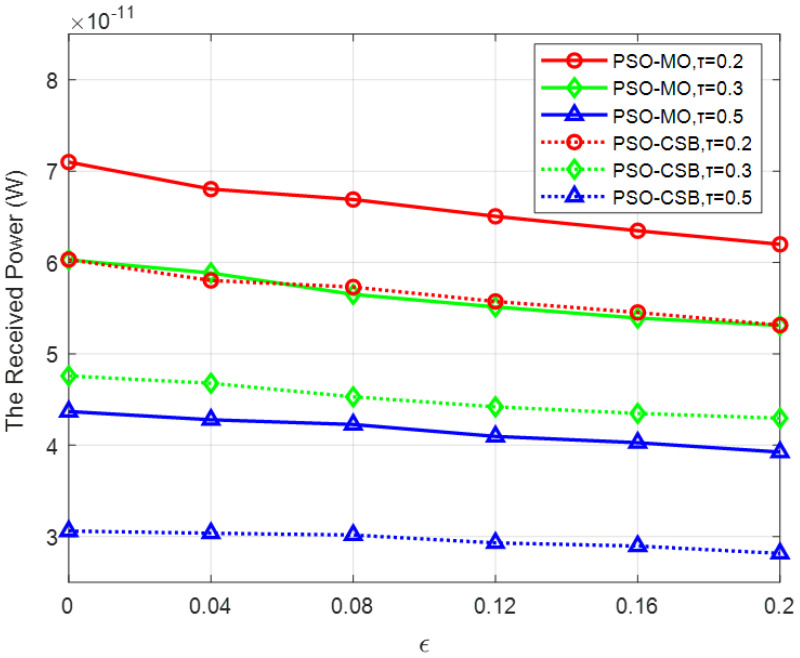
Received power versus the imperfect CSI factor ϵ.

In Figure 11, we analyze the impact of imperfect CSI on the proposed PSO-MO and PSO-CSB algorithms under different sparsity factors τ. To model the imperfect CSI, we define an imperfect CSI factor ϵ, which affects both the BS–IRS channel h and the IRS–User channel $g_{n}$. Specifically, the imperfect channels are expressed as $\hat{h}=h+\sqrt{\epsilon }\alpha z_{0}$, and $\hat{g_{n}}=g_{n}+\sqrt{\epsilon }\beta z_{n}$, where $z_{i}\sim CN(0,1),\;i=0,1,\ldots ,N.$ From the simulation results, it can be observed that as ϵ increases from 0 to 0.2, the received power for both the PSO-MO and PSO-CSB algorithms decreases. This decline becomes more significant as the CSI accuracy deteriorates. Nevertheless, both the PSO-MO and PSO-CSB algorithms exhibit robustness to imperfect CSI. It is particularly noteworthy that when the sparsity factor τ is larger (i.e., when the number of STEs increases), the performance degradation of both algorithms slows down. This phenomenon can be attributed to the fact that the phase shift optimization of the STEs considers multiple potential users within the angular space, whereas the phase shifts of the DTEs are optimized specifically for the current user’s location. This distinction further highlights the robustness of our proposed algorithms in scenarios with imperfect CSI.

## 6. Conclusions

In this paper, we investigated the average received power maximization problem in an HE-IRS-aided mmWave communication system, where the element position and phase shifts of both the DTEs and the STEs were optimized. To solve this problem, we proposed a PSO-MO algorithm for the element position optimization. Then, we decomposed the optimization of the phase shifts of the DTEs and STEs and alternately solved them. Specifically, we utilized MO to optimize the phase shifts of the STEs and derived a closed-form solution for the phase shifts of the DTEs. In addition, we also proposed a low-complexity iteration-free IRS design method by optimizing the lower bound according to the Cauchy–Schwarz inequality. The simulation results showed that although the proposed HE-IRS employed far fewer DTEs than the conventional one, the average received power was not significantly reduced with the proposed element position and phase shift optimization algorithms, especially in scenarios such as a small angular space, low controlling quantization, and small-scale arrays of IRSs. In our future work, we will further explore the impact of the spatial arrangement of DTEs and STEs on performance by employing different optimization algorithms and try to extend the element position and phase shift optimization to more complex and realistic scenarios, such as those involving multipath fading or blockage. Additionally, we will continue to explore the joint optimization of the sparsity factor alongside element positions and phase shifts. Furthermore, we will make efforts to investigate other optimization algorithms, such as genetic algorithms or deep learning-based methods, to enhance the optimization of element positions and phase shifts in HE-IRS-assisted wireless systems.

## Figures and Tables

**Table 1:** Simulation parameters.

Parameter	Value
Carrier Frequency $\left(f_{c}\right)$	28 GHz
BS–IRS Distance $\left(d_{BI}\right)$	10 m
IRS–User Distance $\left(d_{IU}\right)$	5 m
Path Loss	61.4 dB at 1 m
Path Loss Exponent	2.1
IRS Size (M)	64
User Angular Space (Azimuth)	$\Delta \theta_{t}=60{}^{\circ}$
User Angular Space (Elevation)	$\zeta_{t}=120{}^{\circ}$
Quantization Bits (b)	1 bit
Noise Power $\left(\sigma^{2}\right)$	−124 dBm
Transmit Power $\left(P_{b}\right)$	20 dBm

## Data Availability

Data are contained within the article.
